# TREM-1 inhibitor specificity and timing of treatment initiation can impact its therapeutic efficacy in cancer and inflammatory disease

**DOI:** 10.3389/fonc.2025.1689771

**Published:** 2025-12-01

**Authors:** Alexander B. Sigalov

**Affiliations:** SignaBlok, Inc, Shrewsbury, MA, United States

**Keywords:** TREM-1 inhibitory SCHOOL peptides, pan-TREM-1 blockade, macrophage-restricted TREM-1 blockade, resolution of inflammation, cancer therapy-induced inflammation, acute and chronic inflammation diseases, drug discovery & development, pancreatic cancer

## Abstract

**Background:**

Triggering receptor expressed on myeloid cells 1 (TREM-1) is a key regulator in inflammation and an emerging therapeutic target in oncology and inflammatory disease.

**Objective:**

This study aims to determine whether broad or macrophage-specific TREM-1 blockade demonstrates distinct therapeutic efficacy and superior outcomes in disease models.

**Methods:**

Ligand-independent TREM-1 inhibitory peptides GF9 and GA31 (the latter in a form of macrophage-targeted lipopeptide complexes, GA31-LPC) were evaluated in animal models of pancreatic cancer, sepsis, pulmonary inflammation, and fibrosis. GF9 inhibits TREM-1 on all TREM-1-expressing cells, while GA31-LPC targets TREM-1 predominantly on macrophages.

**Results:**

In fully immunocompetent mice, GF9 and GA31-LPC alone significantly inhibited pancreatic cancer progression. In combination with anti-PD-L1 therapy, GA31-LPC, but not GF9, overcame cancer resistance to PD-L1 checkpoint blockade and synergized with immunotherapy. In PANC-1 xenograft-bearing athymic nude mice, both GF9 and GA31-LPC increased complete response rate and survival when combined with chemotherapy. The effectiveness of these agents was dependent on the timing of treatment initiation. GF9 was effective only when given with but not after chemotherapy. In contrast, GA31-LPC was effective only when given after but not together with chemotherapy. Inhibitor specificity and treatment timing effects of therapeutic TREM-1 blockade were also observed in sepsis and acute lung injury models, but not in fibrosis.

**Conclusion:**

These findings for the first time demonstrate that both inhibitor specificity and timing of treatment initiation are crucial for therapeutic TREM-1 inhibition. This has significant implications for clinical strategies targeting TREM-1, particularly informing tailored treatment approaches for cancer and inflammatory diseases.

## 1 Introduction

First reported in 2000 (1), triggering receptor expressed on myeloid cells 1 (TREM-1) was initially shown to play a role in sepsis (2). Currently, TREM-1 is well recognized as a key player in cancer (3, 4) and numerous other inflammation-associated diseases and disorders of infectious and non-infectious origin (reviewed in (5–9). Upon inflammation, TREM-1 is upregulated and amplifies inflammatory response by mediating release of proinflammatory cytokines and factors (9–11), functioning as a switch between physiological and pathophysiological inflammatory processes.

In animal models, TREM-1 blockade ameliorates cancer (reviewed in (3, 12, 13), sepsis (reviewed in (14, 15), acute respiratory distress syndrome (ARDS) and other acute lung injuries (16–18), inflammatory bowel disease (11, 19, 20), retinopathy (21), gouty arthritis (22), rheumatoid arthritis (RA) (23–25), atherosclerosis (26, 27), empyema (28), brain and spinal cord injuries (29–34), Parkinson’s disease (35), liver diseases (36–40), renal injury and kidney transplantation (41, 42), pertussis (43), skin fibrosis (44), pulmonary fibrosis (PF) (45), hemorrhagic shock (46), reperfusion injury (47), and thrombosis (48). This implicates TREM-1 as a potential “magic bullet” in the treatment of diseases with underlying inflammatory pathologies.

TREM-1 is mainly expressed on neutrophils, monocytes, and macrophages including monocyte-derived macrophages (1, 9). Different types of cells that express TREM-1 can play different or even opposite roles in the pathogenesis of inflammatory diseases (49–53) depending on the disease and the type and stage of inflammatory response. Thus, it is reasonable to hypothesize that the therapeutic activity and efficacy of broad pan-TREM-1 inhibitors that target TREM-1 on all TREM-1-expressing cells can strongly differ from those of cell-restricted TREM-1 inhibitors that target TREM-1 on a certain type of cells that express TREM-1 (e.g., macrophages). Despite more than two decades of intensive research in the field, this question has never been addressed before mostly, due to the lack of cell-restricted approaches to TREM-1 blockade.

**Figure 1:**
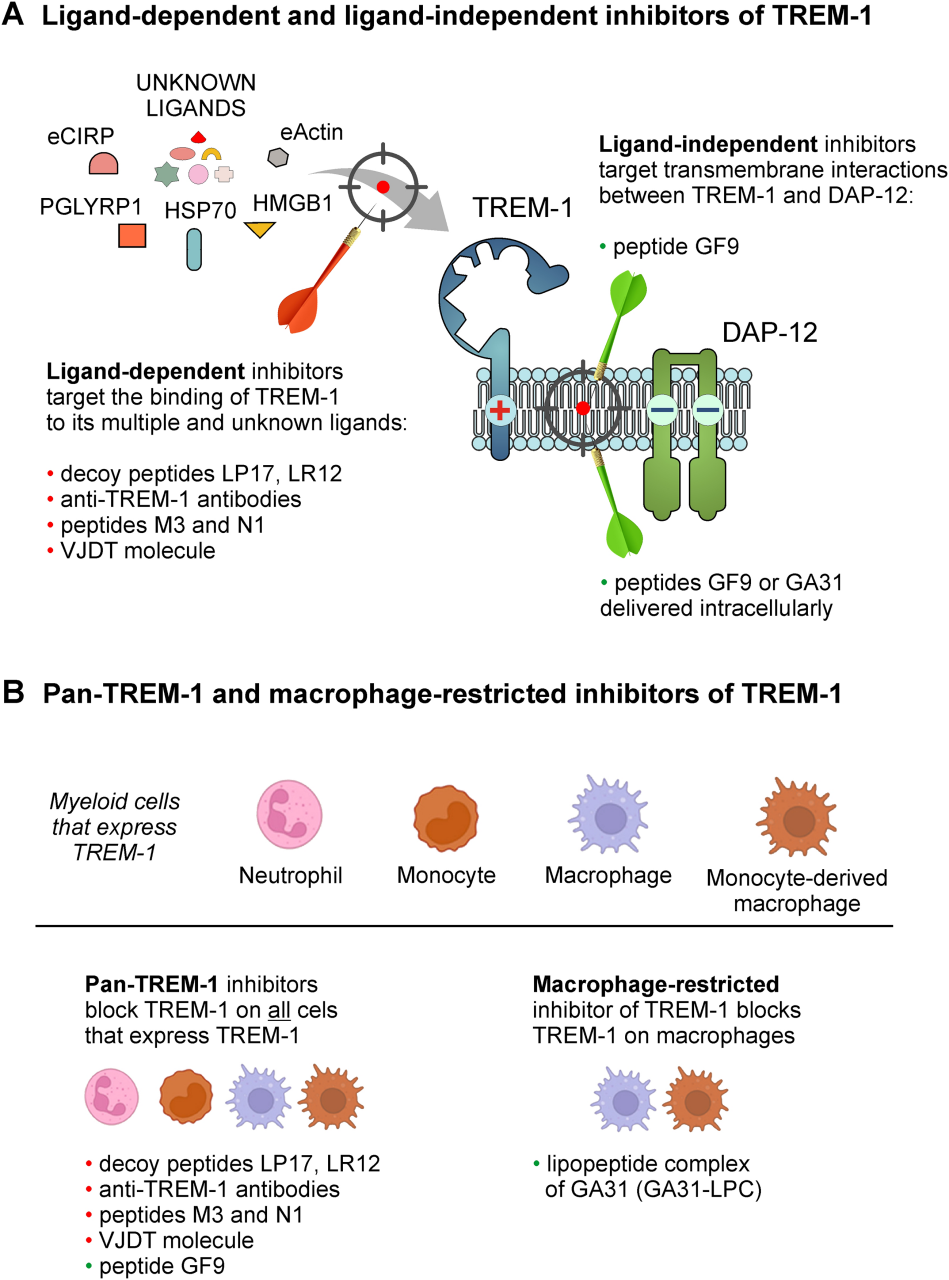
Classification of TREM-1 inhibitors based on their mechanisms of action and cell specificity. **(A)** TREM-1/DAP-12 receptor complex assembly is depicted where TREM-1 and DAP-12 are bound in the cell membrane by electrostatic interactions. Currently available TREM-1 inhibitors can be classified into two groups according to their mechanisms of action: ligand-dependent and ligand-independent inhibitors. Ligand-dependent inhibitors attempt to block interaction of TREM-1 with its multiple and unknown ligands (shown by red arrow), while ligand-independent inhibitors disrupt the interactions between TREM-1 and DAP-12 in the cell membrane (shown by green arrow). **(B)** Based on their cell specificity, TREM-1 inhibitors can be grouped into two families: those that inhibit TREM-1 on all TREM-1-expressing cells (broad or pan-TREM-1 inhibitors) and those that inhibit TREM-1 on a certain type of TREM-1-expressing cells (cell-restricted TREM-1 inhibitors; for example, macrophage-restricted TREM-1 inhibitor). PGLYRP1, peptidoglycan recognition protein 1; eCIRP, extracellular cold-inducible RNA-binding protein; HMGB1, high mobility group box 1; Hsp70, heat shock protein 70 kDa.

Current TREM-1 inhibitors can be broadly classified into ligand-dependent and ligand-independent based on their mechanism of action (**Figure 1A**). Ligand-dependent inhibitors all attempt to block interactions of TREM-1 with its multiple known and unknown ligands by binding either to TREM-1 ligands or to the receptor itself (**Figure 1A**) (54). Examples of ligand-dependent inhibitors that bind to TREM-1 ligands include decoy peptides LP17 and LR12, while anti-TREM-1 monoclonal antibodies (mABs), human eCIRP-derived peptide M3, PGLYRP1 protein-derived peptide N1 and small molecule VJDT represent ligand-dependent inhibitors that bind to TREM-1 (**Figure 1A**). In terms of cell specificity, ligand-dependent inhibitors (**Figure 1A**) are all broad (pan-TREM-1) inhibitors since they inhibit TREM-1 on all cells that express TREM-1 (**Figure 1B**).

Ligand-independent approach to TREM-1 blockade is principally different and uses structural features of TREM-1. TREM-1 has no intracellular signaling domain and signals via its partner, DAP12 (1, 55) (**Figure 1A**). As such, TREM-1 belongs to the family of the so-called multichain immune recognition receptors (MIRRs) with ligand-recognizing and signal-transducing domains located on separate receptor subunits (chains) (56). First reported in 2004 (56, 57), Signaling Chain HOmoOLigomerization (SCHOOL)-driven molecular mechanisms of MIRR signaling revealed the transmembrane protein-protein interactions between MIRR ligand-recognizing and signal-transducing subunits as therapeutic targets that can be targeted by short synthetic peptides (SCHOOL peptides) (56, 58, 59). When applied to TREM-1, this led to discovery of the TREM-1 inhibitory SCHOOL peptide sequence GF9 that inhibits TREM-1 by disrupting the interactions between TREM-1 and DAP-12 in the membrane (**Figure 1A**). This inhibitory action has been shown to ameliorate cancer and many other inflammatory diseases and conditions *in vivo* (25, 44, 59–63).

Molecular mechanisms of ligand-independent TREM-1 blockade allow to develop not only pan-TREM-1 but also cell-specific TREM-1 inhibitors. When systemically delivered as a free peptide, GF9 reaches its site of action in the cell membrane from outside the cell (**Figure 1A**). GF9 lacks cell specificity and inhibits TREM-1 on all cells that express TREM-1 and as such, represents a pan-TREM-1 inhibitor (**Figure 1B**). To develop a macrophage-restricted TREM-1 inhibitor, the peptide GA31 containing the peptide sequence GF9 at the N-terminal end has been designed and incorporated into macrophage-specific nanosized lipopeptide complexes, GA31-LPC (25, 59). When intracellularly delivered by GA31-LPC to macrophages via scavenger receptor A (SR-A)-mediated endocytosis (36), GA31 is released and reaches its site of action in the cell membrane from inside the cell (**Figure 1A**). Thus, by inhibiting TREM-1 predominantly on macrophages, GA31-LPC represents a macrophage-restricted TREM-1 inhibitor (**Figure 1B**).

Here, GF9 and GA31-LPC were used to comparatively study pan-TREM-1 and macrophage-restricted TREM-1 blockades in animal models of pancreatic cancer, sepsis, pulmonary inflammation and fibrosis. The findings reported here demonstrate for the first time that efficacy of pharmacological TREM-1 blockade critically depends on inhibitor specificity and timing of treatment initiation as well as on type of the disease and the animal model used. This has significant implications for clinical strategies targeting TREM-1, particularly informing tailored treatment approaches for cancer and inflammatory diseases. A lack of consideration of this previously unrecognized phenomenon may cause serious misinterpretations of data of preclinical and clinical studies evaluating the efficacy of inflammation-targeting strategies including discovery and development of therapeutic modulators of TREM-1 signaling.

## 2 Materials and methods

### 2.1 TREM-1 inhibitory peptides and formulations

Previously reported TREM-1 inhibitory peptides GF9 and GA31 (25, 44, 59) were synthesized by AmbioPharm, Inc. (North Augusta, SC, USA). Macrophage-specific LPC formulations containing GA31 were generated essentially as previously reported (25).

### 2.2 *In vivo* mouse studies

Animal experiments were performed by various contract research organizations (CROs), including Translational Drug Development (TD2; Scottsdale, AZ, USA), Washington Biotechnology, Inc. (WBI; Baltimore, MD, USA), Crown Bioscience (San Diego, CA, USA), Noble Life Sciences (Sykesville, MD, USA), Charles River Laboratories (Wilmington, MA, USA), and the Center for Translational Medicine at Thomas Jefferson University (Philadelphia, PA, USA) on a fee-for-service basis.

#### 2.2.1 Cancer

In xenografts studies in female athymic nude mice (Crl: NU(NCr)-Foxn1^nu^), the mice were inoculated subcutaneously in the right flank with 0.1 mL of a 50% DMEM/50% Phenol Red-free Matrigel mixture containing a suspension of 5 x 10^6^ cells/mouse of PANC-1 tumor cells. Twenty-six days following inoculation, mice with tumor volumes of 71–159 mm^3^ were randomized into groups of ten mice, each with a group mean tumor volume of 107–108 mm^3^ by random equilibration. PANC-1 xenograft-bearing mice were treated intraperitoneally (i.p.) with vehicle (phosphate-buffered saline, PBS, pH 7.4), 25 mg/kg GF9 or 13 mg/kg GA31-LPC in combination with standard-of-care (SOC) chemotherapy (80 mg/kg gemcitabine i.p. Q3Dx4 and 30 mg/kg nanoparticle albumin based paclitaxel (nab-ptx) intravenously, i.v., Q3Dx4) starting either Day 1 or Day 13 and then continuing as post-chemo therapy (QDx7 every other week to the end of study at Day 99). Tumor volumes and body weights were recorded when the mice were randomized and two times weekly thereafter. The relative tumor volume (RTV) was calculated using the following formula: RTV = (tumor volume on measured day)/(tumor volume on day 0). All results were expressed as the median values (n = 10). Complete response was identified as tumor volume of less than 13.5 mm^3^ for three consecutive measurements. The study was terminated on Day 99 and the remaining mice were sacrificed by regulated CO_2_.

In syngeneic orthotopic studies in female C57BL/6 wild-type (WT) mice, the mice (n = 10 per group) were anesthetized by i.p. injection of 50 mg/kg pentobarbital sodium and inoculated into the subcapsular region of the pancreas with a mPA6115-Luc [luciferase-expressing Kras (G12D)/Trp53 null/Pdx1-cre (KPC)] tumor chunk. Mice were IVIS imaged for bioluminescence on Day 3 and assigned to the respective treatment groups based on the bioluminescent photon flux. Mice were dosed within 24 hours of randomization. Treatment with vehicle (PBS, pH 7.4), GF9 (25 mg/kg, once a day for 3 weeks, i.p.) or GA31-LPC (13 mg/kg, once a day for 3 weeks, i.p.) alone and in combination with immunotherapy, an anti-mouse PD-L1 antibody (10 mg/kg, twice a week for 3 weeks, i.p.), was initiated after grouping and continued throughout the experiment. Tumors were measured by bioluminescent imaging twice a week starting Day 4. The study was terminated on Day 23 and the remaining mice were sacrificed by regulated CO_2_.

#### 2.2.2 Sepsis

In endotoxic shock studies in male C57BL/6 WT mice, the mice (n = 10 per group) challenged with lipopolysaccharide (LPS) essentially as described previously (64) were treated i.p. once with vehicle (PBS, pH 7.4), 25 mg/kg GF9 or 13 mg/kg GA31-LPC 1 h prior or 1 or 3 h post LPS. In positive control group, mice were treated i.p. once with 0.1 mg/kg dexamethasone 1 h prior LPS. The study was terminated on Day 8 and the remaining mice were sacrificed by regulated CO_2_.

In cecal slurry injection (CSI) studies in female Balb/C mice, the mice (n = 12 per group) challenged with CSI essentially as described (65) were treated i.p. once with vehicle (PBS, pH 7.4), 25 mg/kg GF9 or 13 mg/kg GA31-LPC 1 h prior or 6 or 12 h post CSI challenge. In positive control group, mice were treated i.p. with 25 mg/kg ceftriaxone and 12.5 mg/kg metronidazole (ABX) preventively 1 h prior CSI, then daily for 5 days. The study was terminated on Day 7 and the remaining mice were sacrificed by regulated CO_2_.

#### 2.2.3 Pulmonary inflammation and fibrosis

In acute lung injury studies in male Sprague Dawley (SD) rats, the rats (n = 10 per group) were treated with LPS from E.coli O26:B6 dosed via oropharyngeal aspiration (o.p.) once on Day 1 (0 hr). In the prevention model, rats were dosed i.p. once with vehicle (PBS, pH 7.4), 25 mg/kg GF9 or 13 mg/kg GA31-LPC 1 h prior LPS. In the treatment model, rats were dosed i.p. once with 25 mg/kg GF9 or 13 mg/kg GA31-LPC 1 h post LPS challenge. All animals were euthanized 6 h post LPS challenge by lethal i.p. injection of 100 mg/kg pentobarbitone followed by exsanguination. Lungs were lavaged with sterile PBS (without calcium and magnesium) to collect bronchoalveolar lavage fluid (BALF). BALF (per animal) were weighed and total and differential BALF cell counts were determined in cell pellets.

In PF studies in C57BL/6 WT mice, the mice (n = 16 per group) were treated with bleomycin (BLM) intratracheally (i.t.) once on Day 0. In the prevention model, mice were dosed i.p. daily with vehicle (PBS, pH 7.4), 25 mg/kg GF9 or 13 mg/kg GA31-LPC for two weeks starting Day 1. At Day 7, half of mice from each group were sacrificed. In the treatment model, mice were dosed i.p. daily with vehicle (PBS, pH 7.4) 25 mg/kg GF9 or 13 mg/kg GA31-LPC for two weeks starting Day 15. After euthanization by regulated CO_2_ (Days 7 and 22 in the prevention model or Day 28 in the treatment model), the left lungs were lavaged with sterile PBS to collect BALF. The right lungs were harvested and analyzed for lung hydroxyproline.

### 2.3 Statistical analysis

For comparative analyses between two groups of data, statistically significant differences were assessed by Student´s unpaired t-test for normally distributed variables. When the assumption of Gaussian distribution was not met, a non-parametric Mann-Whitney U-test was used for comparisons. For comparisons between more than two groups, statistical differences were analyzed with the one- or two-way analysis of variance (ANOVA) followed by Bonferroni *post hoc* test. Kaplan-Meier survival curves were analyzed using the log-rank test. A p-value < 0.05 was considered statistically significant: *p < 0.05; ** p < 0.01; *** p < 0.001 and **** p < 0.0001. Statistical calculations and graphs were done using GraphPad Prism software.

## 3 Results

### 3.1 Anti-tumor efficacy of GF9 and GA31-LPC oppositely depends on the timing of treatment initiation relative to chemotherapy in cancer mice with intact innate immunity but lacking T cells

Similar to other cancer treatments (e.g., surgery, radiation, radiopharmaceuticals, antibody-drug conjugates, etc.) (66–69), current SOC chemotherapies, including gemcitabine/nab-ptx and FOLFIRINOX (68), represent a double-edged sword: they reduce tumor burden by killing cancer cells, however, the resulting dead tumor cells, or debris, induce inflammation that may lead to the failure of therapy, cancer recurrence and metastasis (66, 69–71). This suggests that timely resolution of therapy-induced inflammation may improve response to treatment as well as prevent tumor recurrence and metastasis resulting in improving quality of life and overall survival of patients.

In this study, to demonstrate that dampening of the TREM-1-mediated inflammation induced by cancer treatment can suppress cancer progression and improve response rate, the anti-tumor efficacy of GF9 and GA31-LPC and its dependence on treatment timing relative to chemotherapy were evaluated in subcutaneous PANC-1 xenograft-bearing athymic nude mice. Selection of this model was based on two considerations: (a) TREM-1 is involved in innate immunity (6), and (b) athymic nude mice retain a fully functional innate immune system (natural killer cells, macrophages, neutrophils) but lack mature T cells (72), which makes the model especially suitable for studying modulators of innate immunity such as TREM-1 inhibitors.

**Figure 2:**
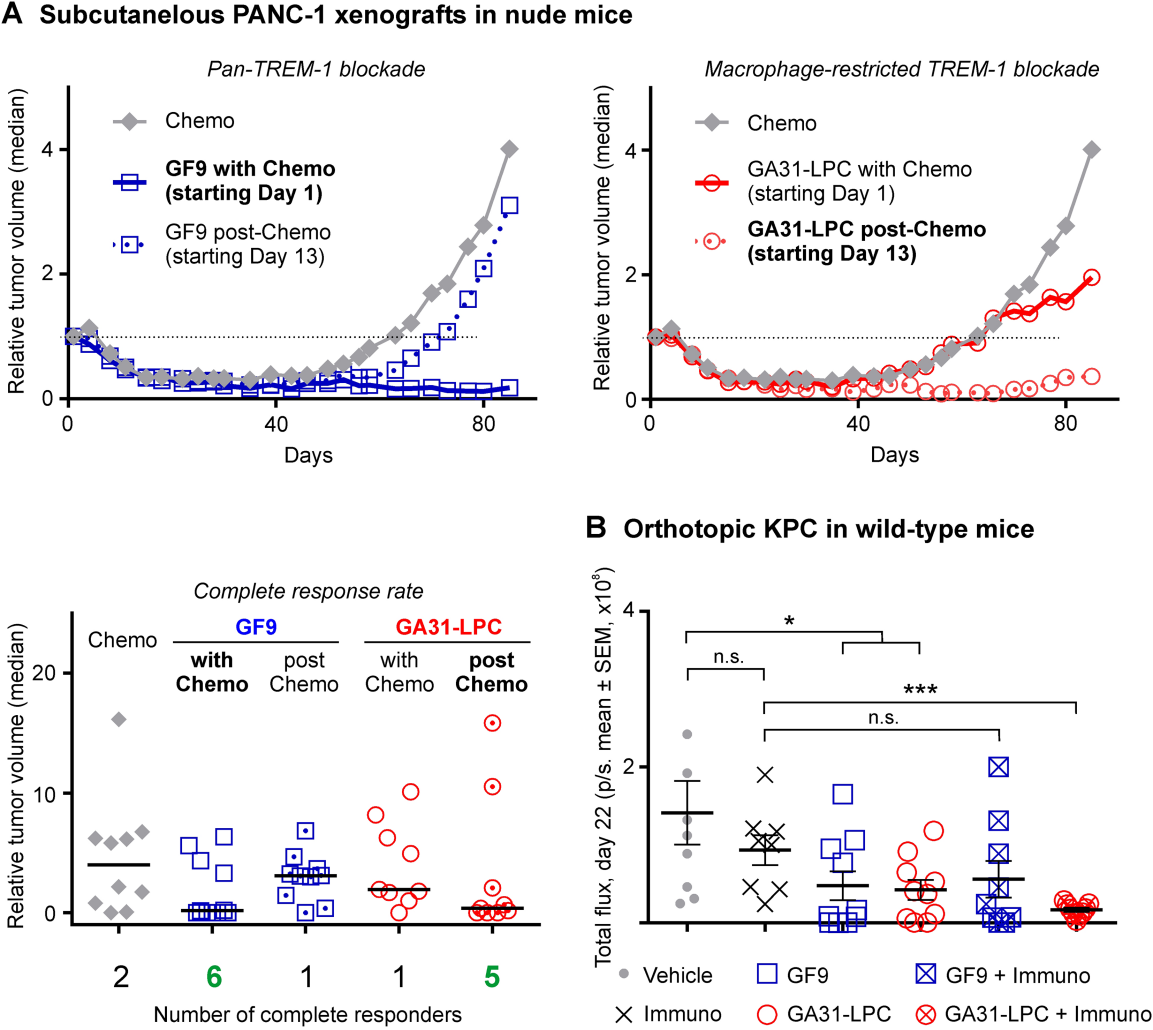
Comparative anti-tumor efficacy of GF9 and GA31-LPC alone and in combination with chemotherapy or immune checkpoint blockade. **(A)** In athymic nude mice, GF9 significantly inhibited PANC-1 tumor growth and improved the complete response rate when given with but not post-chemotherapy. In contrast, GA31-LPC was effective when given post-chemotherapy but not with it. The relative tumor volume (RTV) was calculated using the following formula: RTV = (tumor volume on measured day)/(tumor volume on day 0). The study was terminated on Day 99. All results are expressed as the median values (n = 10). Complete response was identified as tumor volume of less than 13.5 mm^3^ for three consecutive measurements. **(B)** In fully immunocompetent mice, GA31-LPC and GF9 alone both inhibited orthotopic KPC tumor growth. In combination with PD-L1 blockade, GA31-LPC but not GF9 overcame tumor resistance and synergized with immunotherapy. Tumors were measured by IVIS bioluminescent imaging twice a week starting Day 4. The study was terminated on Day 23. KPC, luciferase-expressing Kras (G12D)/Trp53 null/Pdx1-cre tumor. *p<0.05; ***p<0.01; n.s., not significant.

In combination with chemotherapy, both GF9 and GA31-LPC synergistically inhibited tumor growth, improved survival (not shown) and up to three times increased the complete response rate as compared to chemotherapy alone (**Figure 2A**). However, GF9 and GA31-LPC exhibited opposite dependence of the efficacy outcomes on the timing of treatment initiation relative to chemotherapy (**Figure 2A**). While GF9 was effective when given with but not after chemotherapy, GA31-LPC was effective only when given after chemotherapy but not together with chemotherapy. As in our previous studies in mice bearing other pancreatic cancer xenografts (73), ligand-independent TREM-1 blockade by using GF9 and GA31-LPC was well tolerable in this study (data not shown).

In summary, these findings indicate that combination of chemotherapy with broad (pan-TREM-1) or macrophage-restricted TREM-1 blockade yielded a significant synergistic anti-tumor effect compared to chemotherapy alone. The observed therapeutic efficacy and outcome critically depended on the TREM-1 inhibitor specificity and the timing of treatment initiation relative to chemotherapy. Together, these data provide the first experimental *in vivo* evidence that inhibitor specificity and treatment timing can significantly impact the anti-tumor effect of TREM-1-mediated modulation of the innate immune response caused by cancer therapy.

### 3.2 GA31-LPC but not GF9 overcomes tumor resistance to PD-L1 blockade and synergizes with immunotherapy in fully immunocompetent cancer mice

To determine whether pan-TREM-1 and macrophage-restricted TREM-1 blockade alone or in combination with immune checkpoint blockade (ICB) are similarly effective against cancer in fully immunocompetent mice, C57BL/6 WT mice with orthotopically inoculated KPC tumor were treated with GF9 or GA31-LPC alone or in combination with anti-PD-L1 antibody.

In this study, both GF9 and GA31-LPC alone significantly inhibited KPC tumor progression (**Figure 2B**). These findings are in line with the literature data which revealed that GF9 alone inhibits tumor growth and improves survival in C57BL/6J WT mice with orthotopically implanted hepatocellular carcinoma (HCC) Hep55.1C (37) and Hepa1.6 (63) tumors.

In agreement with well-known limitations of anti-PD-L1 ICB for the treatment of solid tumors in general (74, 75) and the ineffectiveness of immunotherapy in pancreatic cancer specifically (76), in this study, PD-L1 blockade alone did not affect KPC tumor growth (**Figure 2B**). Treatment of cancer mice with GF9 concurrently with immunotherapy did not exhibit any synergistic anti-tumor activity compared to GF9 alone (**Figure 2B**). In contrast, the combination of PD-L1 blockade with GA31-LPC synergistically suppressed tumor growth compared to GA31-LPC alone (**Figure 2B**).

Thus, these data demonstrate that while TREM-1 blockade alone either with GF9 or GA31-LPC is effective in suppressing tumor progression, only GA31-LPC, but not GF9 could overcome tumor resistance to PD-L1 blockade and synergize with immunotherapy.

### 3.3 GA31-LPC and GF9 differently protect septic mice from death and exhibit opposite dependence on the timing of treatment initiation relative to challenge

Therapeutic effect of TREM-1 blockade in experimental sepsis was first demonstrated in 2001 (2). Currently, TREM-1 is widely recognized as a critical contributor to the immune dysfunction caused by sepsis (14, 15).

To explore whether GF9 and GA31-LPC differ in their ability to protect septic animals against death, two models were used in this study: mice with endotoxic shock induced by LPS and mice with polymicrobial sepsis induced by CSI.

**Figure 3:**
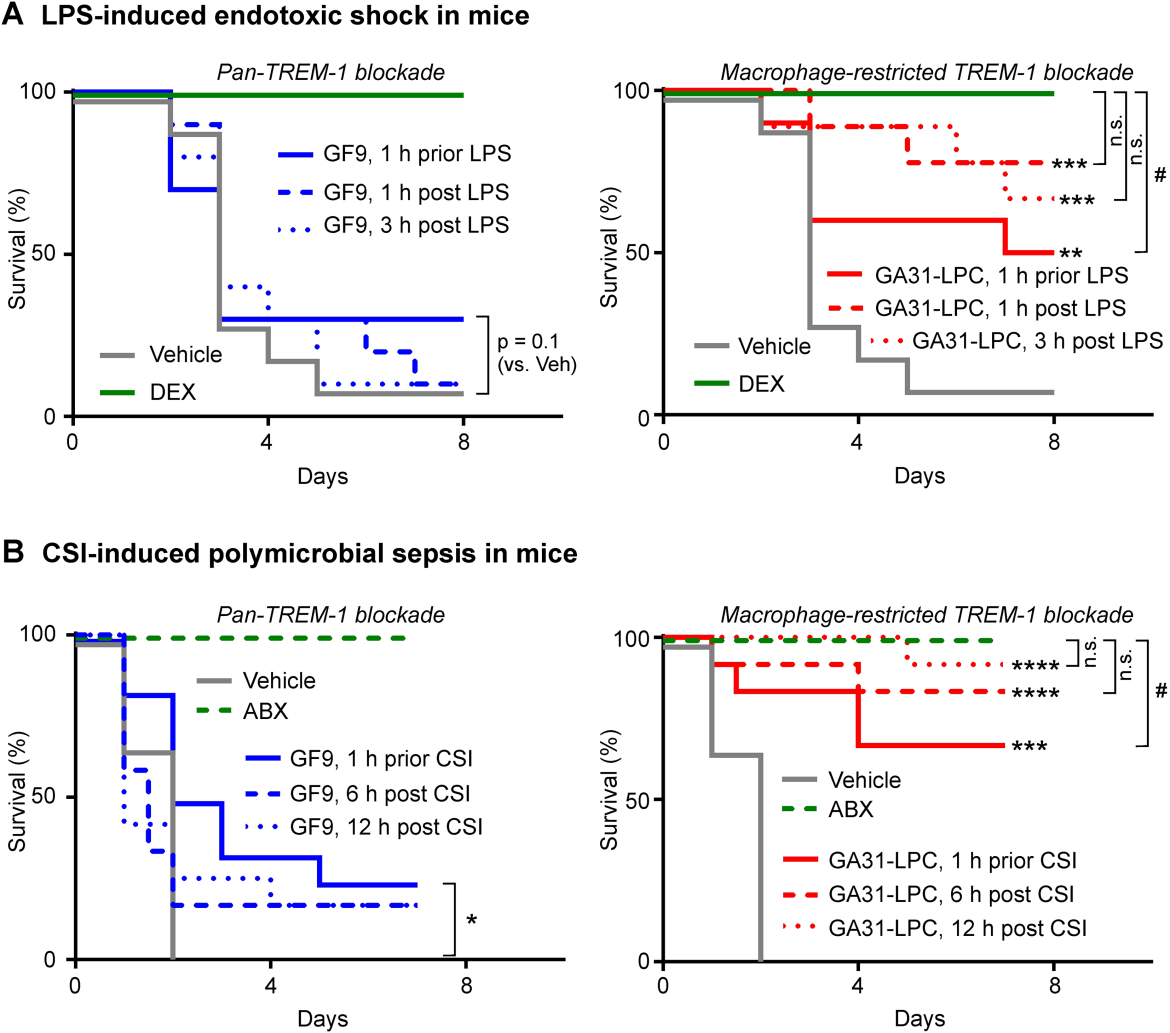
Comparative efficacy of GF9 and GA31-LPC in protecting mice from death caused by endotoxic shock or polymicrobial sepsis. **(A)** In lipopolysaccharide (LPS)-challenged C57BL/6 wild-type (WT) mice, GF9 given once 1 h prior LPS showed a slight but still not significant effect while no effect was observed when GF9 was given once 1 or 3 h post-LPS. In contrast, GA31-LPC significantly protected the animals from death when administered once preventively (1 h prior LPS) and therapeutically (1 or 3 h post LPS). The exhibited efficacy of GA31-LPC given at 1 or 3 h post-LPS did not differ from that shown by positive control (dexamethasone, DEX, 1 h prior LPS) but was paradoxically higher compared to that of GA31-LPC given preventively. **, p<0.01 (versus vehicle); ***, p<0.001 (versus vehicle); ^#^, p<0.05 (versus DEX); n.s., not significant. **(B)** In cecal slurry injection (CSI)-challenged C57BL/6 WT mice, GF9 given once preventively (1 h prior CSI) but not therapeutically (6 or 12 h post-CSI) showed a slight significant effect. In contrast, GA31-LPC significantly protected the animals from death when administered once preventively (1 h prior CSI) and therapeutically at 6 or 12 h post CSI. The exhibited efficacy of GA31-LPC given at 6 or 12 h post-CSI challenge did not differ from that shown by positive control (ceftriaxone and metronidazole, ABX, beginning 1 h prior CSI, then daily for 5 days) but was paradoxically higher compared to that of GA31-LPC given preventively. *p<0.05 (versus vehicle); ***p<0.001 (versus vehicle); ****p<0.0001 (versus vehicle); ^#^p<0.05 (versus ABX); n.s., not significant.

In endotoxemic mice, a slight but still not significant (p=0.1) effect on the survival rate was observed in animals dosed once with GF9 preventively (1 h prior LPS), while therapeutic administration of GF9 (1 or 3 h post LPS) seemingly did not affect animal survival (**Figure 3A**). These findings are in line with the previously observed decrease of effectiveness of TREM-1 blockade in experimental sepsis at later times of treatment with the highest level of protection in the prophylactic animal models (2). In contrast to GF9, GA31-LPC significantly protected the animals from death when administered once either preventively 1 h prior LPS or therapeutically 1 h or 3 h post LPS (**Figure 3A**). The exhibited efficacy of GA31-LPC surprisingly did not decline at later times of treatment (1 or 3 h post-LPS challenge) but rose paradoxically compared to that of GA31-LPC administered preventively (1 h prior LPS) (**Figure 3A**). Further, no significant differences in the survival rate were noted between mice treated therapeutically with GA31-LPC and mice treated preventively with dexamethasone (positive control) (**Figure 3A**).

In line with the data in LPS-challenged endotoxic mice, in mice with polymicrobial sepsis induced by CSI, a slight but significant (p<0.05) effect on the survival rate was observed in animals dosed once with GF9 preventively (1 h prior CSI), while therapeutic administration of GF9 (6 or 12 h post CSI) did not significantly affect animal survival (**Figure 3B**). In contrast, GA31-LPC strongly protected the animals from death when administered once either 1 h prior or 6 or 12 h post CSI (**Figure 3B**). Similar to the data in endotoxemic mice, the exhibited efficacy of GA31-LPC did not decline at later times of treatment (6 or 12 h post-CSI) but rose compared to that of GA31-LPC administered preventively (1 h prior CSI) (**Figure 3B**). Further, no significant differences in the survival rate were noted between mice treated 6 or 12 h post CSI with GA31-LPC and mice preventively treated with ceftriaxone and metronidazole (positive control) beginning 1 h prior CSI, then daily for 5 days.

In summary, these data highlight a marked contrast between pan-TREM-1 and macrophage-restricted TREM-1 inhibitors in their ability to protect endotoxemic or septic mice from death. Notably, the data also reveal a paradoxical enhancement in the therapeutic activity of GA31-LPC when administered as a treatment rather than as a preventative measure. Collectively, these findings strongly support further development of this promising therapeutic approach to significantly improve survival of septic patients.

### 3.4 GA31-LPC but not GF9 significantly suppresses acute pulmonary neutrophilia in rats

Neutrophil infiltration into the lung is a hallmark of acute lung injury (ALI) and its most severe manifestation, ARDS (77). In animal models of LPS-induced ALI, TREM-1 blockade ablates neutrophilic lung inflammation (16, 17).

**Figure 4:**
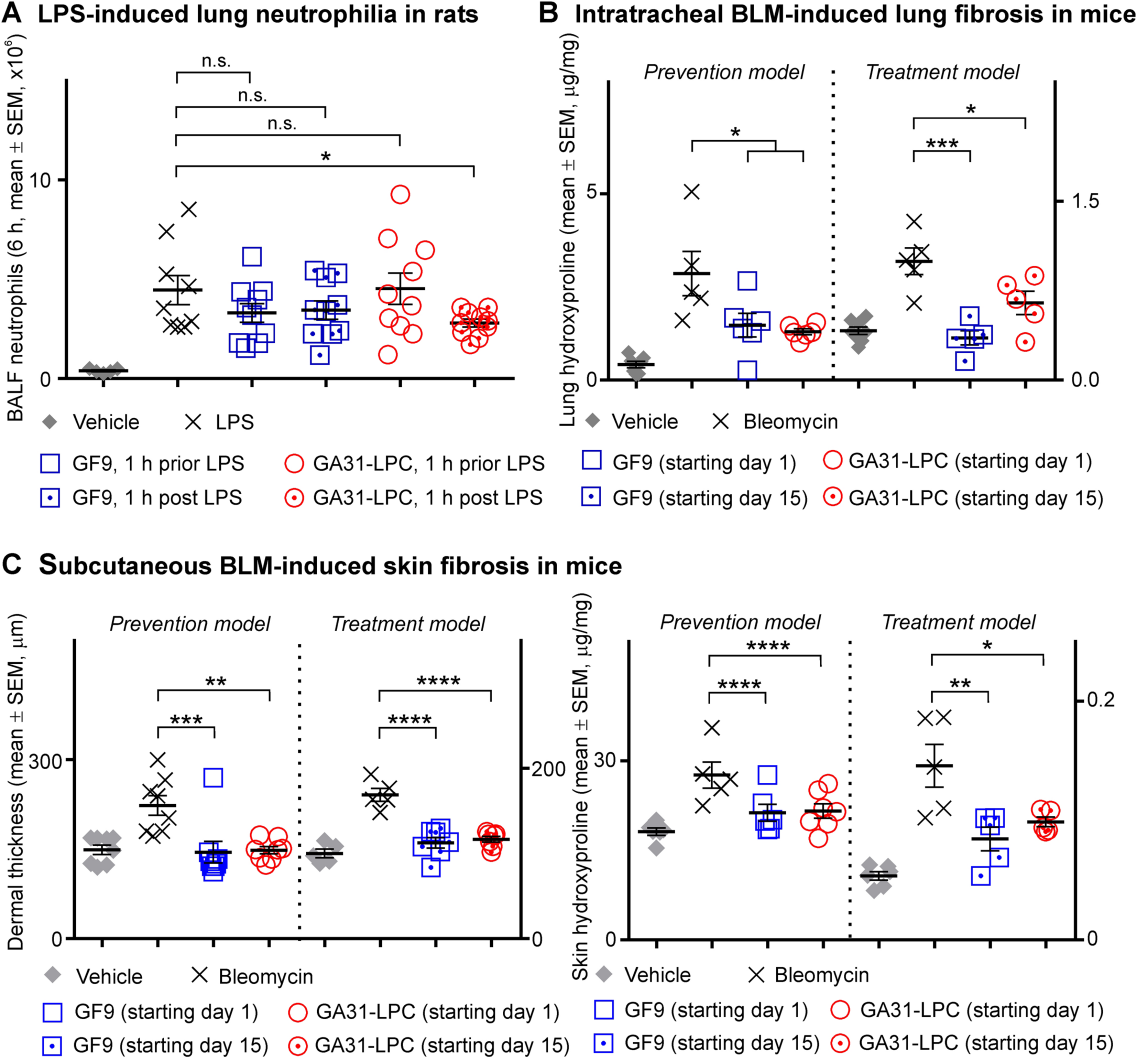
Comparative efficacy of GF9 and GA31-LPC in preventing and treating lipopolysaccharide-induced acute lung injury and bleomycin-induced pulmonary and skin fibrosis. **(A)** In Sprague Dawley (SD) rats with acute lung neutrophilia induced by oropharyngeally aspirated lipopolysaccharide (LPS), GF9 did not affect bronchoalveolar lavage fluid (BALF) neutrophil content when given either preventively (1 h prior LPS) or therapeutically (1 h post LPS). In contrast, GA31-LPC significantly decreased neutrophil infiltration into the lungs when given as a therapeutic (1 h post LPS), but surprisingly not as a preventive (1 h prior LPS) agent. **(B)** In C57BL/6 wild-type (WT) mice with intratracheal bleomycin (BLM)-induced pulmonary fibrosis, GF9 and GA31-LPC were similarly effective in preventing and reversing fibrosis as analyzed by lung hydroxyproline. **(C)** In C57BL/6 WT mice with subcutaneous BLM-induced skin fibrosis, GF9 and GA31-LPC were similarly effective in preventing and reversing fibrosis as analyzed by dermal thickness (left panel) and skin hydroxyproline (right panel). Adapted from Bale S, Verma P, Yalavarthi B, Bajzelj M, Hasan SA, Silverman JN, et al. JCI Insight (2024) 9:e176319. *p<0.05; **p<0.01; ***p<0.001; ****p<0.0001; n.s., not significant.

In this study, a strong dependence of TREM-1 inhibition efficacy on inhibitor specificity and timing of treatment initiation relative to challenge was observed in experimental sepsis (**Figure 3**). Thus, it was reasonable to hypothesize that this may also be the case in experimental ALI. The data generated in rats with LPS-induced pulmonary neutrophilia strongly supported the hypothesis (**Figure 4A**). GF9 did not affect BALF neutrophil content when administered either 1 h prior LPS or 1 h post LPS (**Figure 4A**). In contrast, GA31-LPC significantly decreased neutrophil infiltration into the lungs when given therapeutically (1 h post LPS) but not preventively (1 h prior LPS) (**Figure 4A**). While surprising, this finding is consistent with the data obtained in experimental sepsis that showed superior efficacy of GA31-LPC administered as curative rather than preventive treatment (**Figure 3**).

Thus, these findings mirror the trend observed in this study in animal models of pancreatic cancer, endotoxemia and sepsis, which implies that TREM-1 inhibitor specificity and timing of treatment initiation can strongly impact its therapeutic efficacy in cancer and inflammatory disease.

### 3.5 GF9 and GA31-LPC both effectively prevent and reverse pulmonary fibrosis in mice

TREM-1 plays a role in PF (78) and is overexpressed in the lungs of mice with BLM-induced PF (79). In this study, pan-TREM-1 and macrophage-restricted TREM-1 inhibitors were comparatively tested for prevention and/or treatment of PF induced by intratracheal BLM in mice.

In the prevention model, GF9 and GA31-LPC both effectively prevented PF at Day 22 (**Figure 4B**). At Day 7, GF9 but not GA31-LPC demonstrated inhibition of the earlier fibrotic process (not shown). In the treatment model, GF9 and GA31-LPC both effectively reversed PF (**Figure 4B**). Interestingly, these data are in line with our recently published findings that TREM-1 blockade by either GF9 or GA31-LPC effectively prevented and reversed skin fibrosis induced by subcutaneous BLM in mice (44) (**Figure 4C**, adapted for comparison purposes). Both GF9 and GA31-LPC were demonstrated to mitigate constitutive collagen synthesis and myofibroblast features in systemic sclerosis fibroblasts *in vitro* (44).

Thus, GF9 and GA31-LPC were found to be similarly effective in preventing and treating experimental PF. These findings are consistent with our previous data in experimental skin fibrosis (44) and RA (24, 25), but contrast with the data obtained in this study in animal models of pancreatic cancer, sepsis and acute pulmonary neutrophilia. This is likely to be explained by the chronic nature of PF, skin fibrosis and RA.

## 4 Discussion

Myeloid cells, including neutrophils, monocytes and macrophages, are key players in the innate immunity response but may play dual roles in inflammation and diseases depending on the stage and context of the disease process (52, 53). These closely interacting cells complement each other in the course of defending the host against pathogens and regulating the inflammatory response (52). However, if not tightly regulated, the interactions between myeloid cells can be detrimental to the host by altering the balance between physiological and pathophysiological inflammatory responses (52). Myeloid cell function is closely controlled by TREM receptors including TREM-1 that amplifies the inflammation (2, 9).

Despite growing recognition of TREM-1 as an emerging target in cancer (3, 4) and other inflammatory diseases and disorders (reviewed in (5–9), the question whether pan-TREM-1 and cell-restricted TREM-1 (e.g., macrophage-restricted) inhibitors can differ in their therapeutic potential has never been addressed mainly due to the lack of proper methodology. Previously, we combined the ligand-independent (SCHOOL) mechanism of TREM-1 inhibition by using the peptide sequence GF9 (**Figure 1A**) (56, 59) with a nature-inspired LPC-based technique for delivery of drugs and imaging agents to macrophages via SR-A-mediated endocytosis (36, 56, 59, 80, 81). This resulted in development of a first-in-class macrophage-restricted TREM-1 inhibitor GA31-LPC (**Figure 1B**). While in contrast to tissue-resident macrophages, neutrophils and monocytes do not express SR-A, the expression of SR-A in monocytes is induced during their differentiation to macrophages (82). This suggests that due to the SR-A-mediated mechanism of delivery (36), GA31-LPC will inhibit TREM-1 on both tissue-resident and monocyte-derived types of macrophages, while free peptide GF9 will inhibit TREM-1 on any of TREM-1-expressing cells (**Figure 1B**).

Here, GF9 and GA31-LPC were used to comparatively evaluate their therapeutic efficacy in animal models of pancreatic cancer, sepsis, pulmonary inflammation and fibrosis. To my best knowledge, this is the first study to provide striking experimental evidence for the previously unrecognized dependence of therapeutic efficacy of TREM-1 inhibition on inhibitor specificity, disease, animal model, and timing of treatment initiation.

In cancer, pharmacological blockade of TREM-1 is a promising approach to target tumor-associated macrophages (TAM) (83, 84), the major innate immune cells that infiltrate solid tumors and can form up to 50% of the tumor mass (83). TAMs play a pivotal role in tumor-associated chronic inflammation that promotes tumor progression (85). The clinical significance of TAMs is evidenced by the strong link between TAM number and density and a poor prognosis in 80% of the published cancer studies (86–89). On the other hand, cancer therapy (e.g., surgery, radiation, chemotherapy) can induce a strong acute inflammatory response by causing massive necrotic death of cancer cells and surrounding tissues and triggering a physiological inflammatory reaction similar to a wound-healing response (66, 90). If not resolved, therapy-induced acute inflammation may result in the failure of therapy, cancer recurrence and metastasis (71, 91). However, despite the well-established relationship between inflammation and cancer as well as encouraging data from multiple preclinical and observational population studies, anti-inflammatory therapeutics have failed to demonstrate significant benefit in the treatment or preventing recurrence of cancer (92, 93). One example is canakinumab, a mAb that blocks the proinflammatory cytokine interleukin-1β, that failed in three Phase III trials for non-small cell lung cancer (94, 95).

Collectively, this highlights our poor understanding of the precise mechanisms and nuances involved in the complex relationship between inflammation and cancer, especially regarding different types and stages of inflammation as well as treatment types and timing. One of the key elements here is the balance between inflammation and resolution of inflammation, which if dysregulated, leads to disease pathology that may be associated with maladaptive immunity as a result of unresolved acute inflammation (96). This suggests pro-resolution strategies as a new therapeutic strategy that can be intrinsically more beneficial compared to conventional anti-inflammatory approaches.

In this study, in cancer mice with intact innate immunity but lacking functional adaptive immunity, GA31-LPC but not GF9 demonstrated remarkable anti-tumor activity when given after but not together with chemotherapy (**Figure 2A**). The observed dependence of efficacy on the timing of treatment initiation relative to chemotherapy may reflect the ability of GA31-LPC to resolve acute innate inflammation caused by chemotherapy. Ongoing studies are focused on the determination of the therapeutic window for resolving cancer therapy-induced inflammation by macrophage-restricted TREM-1 blockade. In contrast, GF9 showed significant anti-tumor activity when given with but not after chemotherapy (**Figure 2A**), likely suggesting anti-inflammatory rather than pro-resolving mechanisms of its anti-tumor effect. Synergy of immunotherapy with macrophage-restricted, but not pan-TREM-1 blockade (**Figure 2B**) adds further evidence to support the conclusion on the various contributions of different myeloid cells to cancer progression (97). Further studies in multiple preclinical models of solid tumors are needed for better understanding of the molecular mechanisms of the observed phenomena and their generalizability across different cancer types and treatments.

The hypothesis that macrophage-restricted but not pan-TREM-1 blockade contributes to the resolution of acute inflammatory response, initially originated from the data collected in this research in chemotherapy-treated cancer mice, has been further supported by key findings in animal models of sepsis and ALI. In these models, GA31-LPC demonstrated the striking and seemingly paradoxical dependence of its therapeutic efficacy on the timing of treatment initiation relative to challenge – prophylactic treatment showed the lower or no benefit compared to therapeutic treatment (**Figures 3**, **4A**). Importantly, the observed dependence of the ability of GA31-LPC to protect septic mice from death (**Figure 3**) or inhibit pulmonary neutrophilia in rats (**Figure 4A**) on the timing of treatment initiation is unlikely to be related to the half life of GA31-LPC in circulation since GA31-LPC exhibits biphasic elimination with a rapid phase for 1 h and a slow phase for 40 h (not shown).

Interestingly, both GF9 and GA31-LPC were effective in preventing and treating BLM-induced pulmonary fibrosis (**Figure 4B**), suggesting that anti-inflammatory mechanisms of pan-TREM-1 and macrophage-restricted TREM-1 blockade may importantly contribute to the therapeutic effects in chronic inflammatory diseases. This is in line with the findings in this study that GF9 and GA31-LPC alone show significant anti-tumor efficacy in a fully immunocompetent mouse model of pancreatic cancer (**Figure 2B**). The conclusion is further supported by the data of our previous studies that demonstrated that both GF9 and GA31-LPC effectively prevent and treat such chronic inflammatory diseases as BLM-induced skin fibrosis (**Figure 4C**; adapted from (44)) and collagen-induced arthritis (24, 25).

In summary, this proof-of-concept study is the first to demonstrate the potential use of macrophage-restricted TREM-1 inhibitory strategy for the fine-tuning of the innate immune response by targeting resolution rather than suppression of physiological inflammation in a variety of inflammatory diseases. In cancer, a macrophage-restricted TREM-1 inhibitor could be used in at least, three ways: (a) alone to suppress TAM-mediated chronic inflammation and inhibit cancer progression; (b) in combination with ICB to overcome resistance of hard-to-treat cancers such as pancreatic cancer and synergize with otherwise ineffective immunotherapy, and (c) to complement existing cancer therapies such as surgery, radiation, and chemotherapy and timely resolve the physiological acute inflammatory response caused by these therapies. These approaches can be deployed to prevent cancer recurrence and metastasis, increase the complete response rate, decrease the number of treatment cycles while lengthening intervals between treatments to improve quality of life, and extend overall survival.

## 5 Conclusions

To the best of my knowledge, this is the first time that the efficacy of pharmacological blockade of TREM-1 in animal models of cancer and inflammatory diseases has been demonstrated to depend firstly, on the specificity of TREM-1 inhibitor (pan-TREM-1 or macrophage-restricted TREM-1 inhibitor) and secondly, on the timing of treatment initiation. As TREM-1 has been implicated as an inflammation amplifier involved in virtually all inflammatory conditions, the findings reported here offer novel insights into the intricate and interconnected nature of the physiological and pathophysiological inflammatory immune responses and open new avenues in further research on spatially and temporally distinct roles of myeloid cells in these processes. Further, these findings can be used not only to correctly interpret preclinical and clinical data on TREM-1, but also to design future synergistic therapeutic approaches to overcome challenges of targeting inflammation. This has significant implications for clinical strategies targeting TREM-1, particularly informing tailored treatment approaches for cancer and inflammatory diseases.

## Data Availability

The original contributions presented in the study are included in the article/supplementary material. Further inquiries can be directed to the corresponding author.
